# Effect of alloying in monolayer niobium dichalcogenide superconductors

**DOI:** 10.1038/s41467-022-29213-8

**Published:** 2022-05-02

**Authors:** Darshana Wickramaratne, I. I. Mazin

**Affiliations:** 1 Center for Computational Materials Science, U.S. Naval Research Laboratory, Washington, DC 20375 USA; 2 Department of Physics and Astronomy, George Mason University, Fairfax, VA 22030 USA; 3 Quantum Science and Engineering Center, George Mason University, Fairfax, VA 22030 USA

**Keywords:** Superconducting properties and materials, Electronic properties and materials, Electronic structure

## Abstract

When sulfur and silicon are incorporated in monolayer 2H-NbSe_2_ the superconducting transition temperature, *T*_*c*_, has been found to vary non-monotonically. This was assumed to be a manifestation of fractal superconductivity. Using first-principles calculations, we show that the nonmonotonic dependence of *T*_*c*_ is insufficient evidence for multifractality. A unifying aspect in our study are selenium vacancies in NbSe_2_, which are magnetic pair-breaking defects that we propose can be present in considerable concentrations in as-grown NbSe_2_. We show that sulfur and silicon can occupy the selenium sites and reduce the pair-breaking effect. Furthermore, when sulfur is incorporated in NbSe_2_, the density of states at the Fermi level and the proximity to magnetism in the alloy are both reduced compared to the parent compound. Based on our results, we propose an alternative explanation of the non-monotonic change in *T*_*c*_ which does not require the conjecture of multifractality.

## Abstract (web summary)

The non-monotonic behaviour of the superconducting transition temperature in NbSe2-xSx monolayer alloys has been linked to fractal superconductivity. Here, using first-principles calculations, the authors provide an alternative explanation for this behavior based on the effects of alloying and defects on the electronic structure and magnetism.

## Introduction

Ising superconductivity in two-dimensional materials is a rapidly growing field of theoretical and experimental research^1–8^. The combination of broken-inversion symmetry and strong spin-orbit coupling present in single monolayers (MLs) of the two-dimensional transition metal dichalcogenides leads to Fermi surfaces where the spin of the electrons is perpendicular to the plane of the monolayer and the electron spin direction flips between time-reversal invariant points of the Brillouin zone. This has been experimentally confirmed by establishing, for example in NbSe_2_, that the superconducting critical field is significantly higher in-plane versus out-of-plane, and much larger than the Pauli limit^1^. While there have been extensive phenomenological descriptions of Ising superconductivity, there are several intriguing material-specific puzzles.

In NbSe_2_, which is the most widely studied Ising superconductor, the superconducting transition temperature, *T*_*c*_, decreases from ~6 K to ~3−4 K, when it is reduced from bulk to a single monolayer^1^. Similar studies conducted on NbS_2_ provide an intriguing contrast. In 2H-NbS_2 _*T*_*c*_ is ~6 K, while superconductivity has not been observed in bulk 3R-NbS_2_^9,10^. These two polytypes differ in the stacking of the individual monolayers, while within each ML Nb atoms are in a trigonal prismatic coordination with the chalcogen atom, similar to NbSe_2_. Reducing the thickness of NbS_2_ leads to a strong suppression in *T*_*c*_^11^. Superconductivity has not been found in ML NbS_2_.

It was recently reported that when ML NbSe_2_ is alloyed with sulfur, S, *T*_*c*_ increases up to a S content of *x* = 0.4^12^ in ML NbS_x_Se_2−x_ alloys. For S content greater than ~0.4, *T*_*c*_ was then found to decrease monotonically^12^ exhibiting qualitatively similar behavior to the bulk alloys. A non-monotonic change in *T*_*c*_ was also found when silicon, Si, was deposited on the surface of the same NbSe_2_ samples, where it was assumed that Si was adsorbed on the monolayer. Up to a Si coverage of 0.05 Si atoms per NbSe_2_ formula unit, *T*_*c*_ increased. For larger concentrations of Si, *T*_*c*_ decreased and superconductivity was completely quenched at ~0.17 Si atoms per NbSe_2_ formula unit. These non-monotonic changes in *T*_*c*_ due to S and Si in NbSe_2_ were interpreted as disorder-induced enchancement of *T*_*c*_, which possibly arises from the multifractality of the electronic wave functions^13,14^. Implicit in this assumption is that the effect of alloying (either with Si or S) on electronic and Coulomb interactions is sufficiently weak so as to not impact *T*_*c*_ directly. While this is an enticing consideration, there are several important questions and experimental puzzles that need to be addressed first, which we briefly outline.

**Fig. 1:**
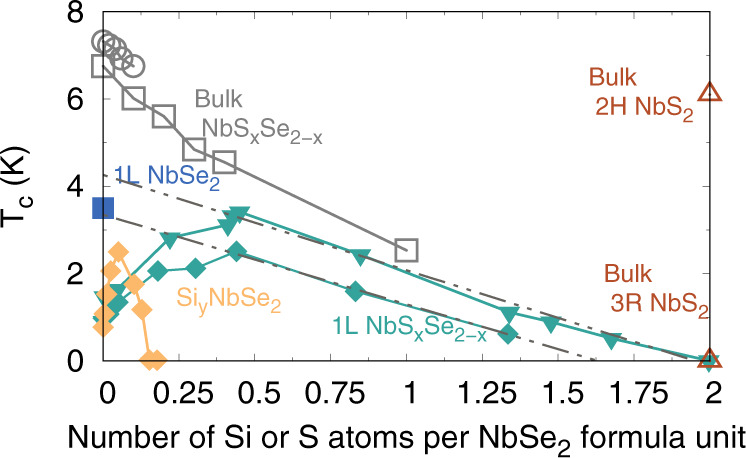
Experimental reports of the superconducting transition temperature versus alloy concentration. Data is for bulk and ML NbS_x_Se_2−x_ alloys as a function of S content, *x* and Si content, *y*. The references associated with each marker is as follows: gray open squares^16^, gray circles^15^, teal and orange filled downward triangles and diamonds^12^, red triangles^10^ and blue filled square^1^. Open symbols correspond to measurements on bulk samples while filled symbols correspond to measurements on ML samples. The dotted lines correspond to a linear extrapolation of the ML data for *x* > 0.4. See the main text for the discussion on the extrapolation.

The measurements where fractal superconductivity was observed report a *T*_*c*_ for ML NbSe_2_ that is ~2 K lower than the widely accepted *T*_*c*_ of ML NbSe_2_, ~3–4 K^1,5^. In fact, the peak *T*_*c*_ where fractal superconductivity is observed is ~3 K, which occurs for 0.2 ≤ *x* ≤ 0.5 due to alloying with S. We also note the experimental in-plane lattice constant is relatively unchanged for 0 ≤ *x* ≤ 0.2 (Supplementary information). If the *T*_*c*_ of NbSe_2_ reported by Zhao et al.^12^ occurred at the more widely accepted 3–4 K, this would not lead to a dome-shaped dependence of *T*_*c*_ on S and Si content, as illustrated in Fig. 1. Instead, *T*_*c*_ would decrease linearly with S content, as has been found when S is alloyed into bulk NbSe_2_^15,16^.

Taken together, we arrive at three possible mechanisms that can lead to this non-monotonic dependence of *T*_*c*_ on S and Si content. The first is the role of fractal superconductivity, which was invoked in Refs. ^12,17^. While this exotic phenomenon may lead to a non-monotonic change in *T*_*c*_^18^, the success of this model requires information on a plethora of material-dependent parameters that are often not accessible by experiment alone. A second possible mechanism is the role of the charge-density wave (CDW), which has been shown to lead to a pseudogapping of the Fermi surface in ML NbSe_2_^19,20^, and thus to a reduction in *T*_*c*_. However, recent studies have suggested the CDW transition temperature varies little when NbSe_2_ transitions from bulk to a single ML^21,22^, while *T*_*c*_ exhibits a large change^1^. This would imply that the coupling between the superconducting and CDW order parameters is weak, as has been found in studies on bulk NbSe_2_^23,24^.

A third mechanism is the collective role of point defects^4^ and spin fluctuations^6^, both of which have been suggested as a source of pair breaking in ML NbSe_2_. Experimental studies on ML NbSe_2_ have found the selenium, Se, vacancy concentration can be large (equivalent to a bulk concentration of ~10^21^ cm^−3^), depending on the growth conditions^25^. Selenium vacancies, which are magnetic point defects in NbSe_2_^26^, can act as a source of pair-breaking and decrease *T*_*c*_. However, during the growth of NbS_x_Se_2−x_ alloys, S, which is isovalent to Se, but more electronegative, can occupy the Se vacancies and lower the concentration of pair-breaking defects. This is analagous to the finding that oxygen can substitute for sulfur (both of which are isovalent) in sulfur-deficient ML TaS_2_, and lead to an increase in *T*_*c*_ compared to ML TaS_2_^27^. Since Si and Se have approximately similar atomic radii the possibility for Si substitution for Se vacancy sites also exists.

Alloying will also lead to changes in the electronic structure, which may also affect the proximity of the material to magnetism or lead to changes in the density of states (DOS) at the Fermi level, and therefore *T*_*c*_. There is a priori no means to determine how all of these properties change with alloying. Furthermore, if defects are indeed the source of the lower *T*_*c*_ in NbSe_2_, this raises questions on the purported relationship between the non-monotonic dependence of *T*_*c*_ and fractal superconductivity^12^.

In the present work we propose an alternative solution that reconciles these puzzles. Using first-principles density functional theory calculations (Sec. Methods) we show that this non-monotonic dependence of *T*_*c*_ on sulfur and silicon content can emerge from the interplay between defects and the effect of alloying on the electronic structure and spin-fluctuations. We show that S is completely miscible in NbSe_2_, across the entire alloy composition range. For finite concentrations of S in NbS_x_Se_2−x_ we find a reduction of the density of states at the Fermi level *and* a weakening of magnetism, compared to the parent compounds, NbSe_2_ and NbS_2_. We also show there is a minimum energy pathway that would result in Si adatoms that are deposited on NbSe_2_ to be incorporated substitutionally on the Se site or as an interstitial. We conjecture a combination of these effects can lead to a non-monotonic dependence of *T*_*c*_ on S and Si content, without having to invoke the phenomenon of multifractality.

## Results

### Predictions for sulfur in NbSe_2_

We start by considering the properties of chalcogen vacancies in NbS_2_ and NbSe_2_ in the dilute limit. The formation energies of a S vacancy, *V*_S_, in NbS_2_ and a Se vacancy, *V*_Se_, in NbSe_2_ is listed in Table 1.

**Table 1:** Formation energy of chalcogen vacancies in NbSe_2_ and NbS_2_ under Nb-rich and Nb-poor conditions.

Defect	Nb-rich (eV)	Nb-poor (eV)
*V*_Se_	0.7	1.7
*V*_S_	1.2	2

The results show that the formation energy of *V*_Se_ is lower than *V*_S_, even under Se-rich conditions that were used in the growth of the NbSe_2_ samples in the study by Zhao et al.^12^. This suggests that as-grown ML NbSe_2_ is likely to have a higher concentration of Se vacancies compared to S vacancies in NbS_2_. We also considered the possibility that S may substitute on the Nb site and calculated the formation energy of this defect, S_Nb_, in ML NbSe_2_. In the dilute limit we find the formation energy of S_Nb_ to be larger than the formation energy of *V*_Se_. Hence, for the purposes of alloying beyond the dilute limit we only consider substitution of S on the Se site.

**Fig. 2:**
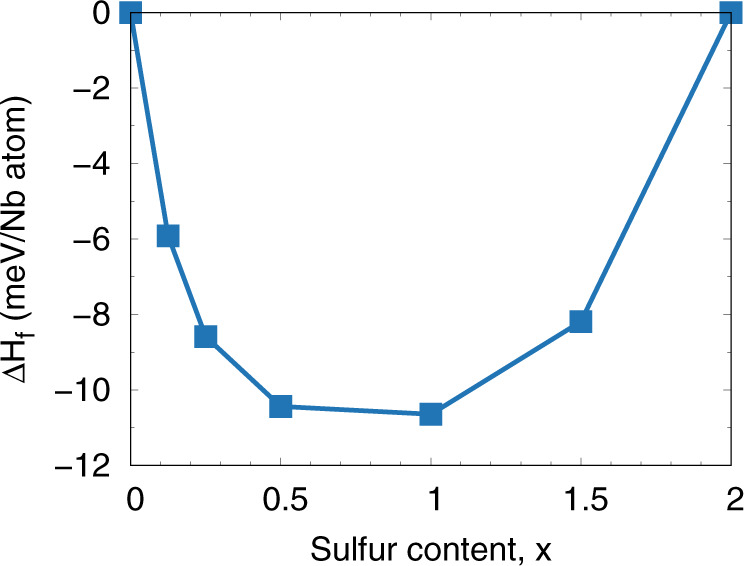
Formation enthalpy as a function of S content, *x*, in NbS_x_Se_2−x_. Calculations are performed using a (4 × 4 × 1) supercell of the monolayer structure.

Next we check the stability of NbS_x_Se_2−x_ alloys with respect to decomposing into their parent compounds, NbSe_2_ and NbS_2_. Figure 2 illustrates the lowest enthalpy structure for each composition. We find the *T* = 0 K formation enthalpy across the entire range of compositions is negative which suggests ordered NbS_x_Se_2−x_ alloys are stable with respect to decomposition into the parent compounds.

We now turn to the electronic and magnetic properties of the alloys. We first consider the parent compounds, NbSe_2_ and NbS_2_. In a single ML the Nb atoms are in a trigonal prismatic coordination with the chalcogen atoms. In ML NbSe_2_ the trigonal crystal field that acts on the 4*d* states of Nb^4+^ leads to one band that crosses the Fermi level, which generates Fermi contours at Γ, K and K${}^{\prime}$^6^. The combination of broken inversion symmetry in the monolayer and strong spin-orbit coupling leads to a spin-orbit splitting of the spin degenerate band along the M-K-Γ line of the Brillouin zone. Since the 4*d* states of Nb^4+^ in NbS_2_ are also in a trigonal prismatic coordination, albeit with a shorter Nb-S bond length compared to the Nb-Se bond length, the qualitative features of the band structure between the two materials are similar (Supplementary information).

ML NbSe_2_ exhibits strong spin fluctuations, which have been highlighted as a potential source of pair breaking^6,28–30^. First-principles calculations have shown that monolayer NbSe_2_ can host ferromagnetic spin fluctuations with a sizeable Stoner renormalization, and an antiferromagnetic spin spiral state with **q** vector (0.2, 0, 0)^6,29^. In ML NbSe_2_ we find the spin spiral state to be 1.7 meV/Nb atom lower in energy compared to the non-magnetic state. In NbS_2_, we find a spin spiral state at a **q**-vector of (0.2, 0, 0) is also stable (Supplementary information) and is is 1.9 meV/Nb atom lower in energy compared to the non-magnetic ground state. If spin fluctuations are sizeable in the alloy they can impact pairing interactions.

**Fig. 3:**
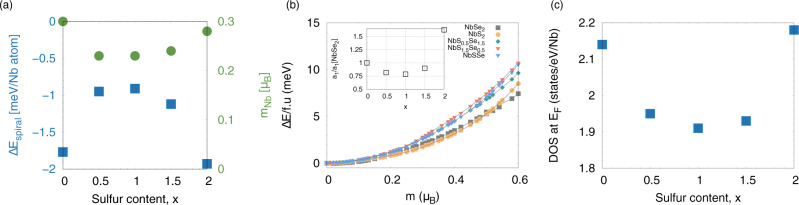
Electronic and magnetic properties of the NbS_x_Se_2−x_ alloys. **a** Energy difference between the spin spiral state and the non magnetic state as a function of sulfur content, *x*, in NbS_x_Se_2−x_ (blue filled squares, left vertical axis). Magnetic moment per Nb atom as a function of sulfur content in the spin spiral calculation with finite *q* (green circles, right vertical axis). **b** Collinear fixed-spin moment calculations of NbSe_2_ (gray squares), NbS_2_ (orange circles), NbS_0.5_Se_1.5_ (teal diamonds), NbSSe (red downward triangles), and NbS_1.5_Se_0.5_ (blue triangles) illustrate the change in energy per formula unit with respect to the non-magnetic state as a function of magnetic moment per Nb atom. The inset illustrates the coefficient *a*_1_ (see main text) normalized by the value of *a*_1_ in NbSe_2_. **c** Density of states at the Fermi level, *E*_*F*_, as a function of sulfur content, *x*. The magnitude of the DOS for NbSe_2_ and NbS_2_ correspond to the spin spiral state (Supplementary information).

To study the effect of alloying on the spin spiral energies we use virtual crystal approximation (VCA) calculations (Sec. Methods) for S contents that correspond to *x* = 0.5, 1 and 1.5. Figure 3a illustrates the energy difference between the spin spiral state with respect to the non-magnetic state, Δ*E*_spiral_, in NbS_x_Se_2−x_ When S is alloyed into NbSe_2_, the spin spiral state is less stable for intermediate values of S content than for either NbSe_2_ and NbS_2_. At *x* = 1 we find Δ*E*_spiral_ decreases by a factor of 2.1 compared to NbS_2_ where the magnitude of Δ*E*_spiral_ is the largest. The magnitude of the magnetic moment on the Nb atom is also suppressed by up to ≃ 25% in the spin-spiral state for the alloys with finite S content compared to the parent compounds, as illustrated in Fig. 3a.

Next we consider whether ferromagnetic spin fluctuations, which are present in NbSe_2_, are also impacted due to alloying by using the VCA and collinear fixed-spin moment calculations for ML NbSe_2_, NbS_2_, NbSSe, NbS_1.5_Se_0.5_, and NbS_0.5_Se_1.5_. The quantity of interest is the ferromagnetic spin susceptibility, *χ*, which is defined as $\chi ={a}_{1}^{-1}={\left(\frac{{\delta }^{2}E}{\delta {m}^{2}}\right)}^{-1}$ (see Methods). We find that  *χ* varies non-monotonically as a function of S content as illustrated in the inset of Fig. 3b, where it is large for NbSe_2_ and NbS_2_ and suppressed in the case of the alloys. Hence, it is reasonable to assume that the spin fluctuations for intermediate concentrations are supressed non-monotonically for all relevant wave vectors. In both cases these fluctuations are the weakest at roughly equal concentrations of S and Se.

The origin of the reduction in *χ* (and, probably, also Δ*E*_spiral_, given the relatively small spiral vector of (0.2, 0, 0)) can be understood by examining the density of states (DOS). The DOS at the Fermi level, *N*(E_F_) as a function of S content is illustrated in Fig. 3c. In NbSe_2_ and NbS_2_, *N*(*E*_*F*_) is suppressed from 2.8 states/eV/Nb atom in the nonmagnetic structure to 2.14 states/eV/Nb atom in NbSe_2_ and 2.18 states/eV/Nb atom in NbS_2_ in the spin spiral ground state (Supplementary information). We also find *N*(*E*_*F*_) is suppressed for the alloys at *x* = 0.5, 1, and 1.5, where in the non-magnetic state *N*(*E*_*F*_) is ~2.5 states/eV/Nb atom while in the spin spiral state it reduces to 1.9 states/eV/Nb atom. Hence, for the parent compounds and the alloys, our calculations indicate there is a gain in one-electron energy by transitioning to the spin spiral state. We also find that in the spin spiral state, the magnitude of *N*(*E*_*F*_) of the alloys decreases by 10% compared to *N*(*E*_*F*_) of the parent compounds. Such a small change in *N*(*E*_*F*_) as a function of S content is consistent with the fact that *N*(*E*_*F*_) is comprised almost entirely of Nb *d*-states in NbSe_2_ and NbS_2_.

### Predictions for silicon in NbSe_2_

We now present our results for the properties of Si in monolayer NbSe_2_. The formation energies for Si incorporated substitutionally (on the Nb site, Si_Nb_ and on the Se site, Si_Se_), adsorbed at high-symmetry positions on top of the NbSe_2_ monolayer, Si_ads_ (where we consider the hollow site formed by the triangle of Se atoms, Si${}_{{{{{{{{\rm{ads}}}}}}}}}^{{{{{{{{\rm{hollow}}}}}}}}}$, and vertically above either a Se atom, Si${}_{{{{{{{{\rm{ads}}}}}}}}}^{{{{{{{{\rm{Se}}}}}}}}}$, or Nb atom, Si${}_{{{{{{{{\rm{ads}}}}}}}}}^{{{{{{{{\rm{Nb}}}}}}}}}$) and Si incorporated interstitially, Se_*i*_, is summarized in Table 2.

**Table 2:** Formation energy of Si in NbSe_2_ under Nb-rich conditions.

Defect	Formation energy (eV)
Si_Nb_	3.04
Si_Se_	1.26
Si_*i*_	1.59
Si${}_{{{{{{{{\rm{ads}}}}}}}}}^{{{{{{{{\rm{hollow}}}}}}}}}$	1.14
Si${}_{{{{{{{{\rm{ads}}}}}}}}}^{{{{{{{{\rm{Se}}}}}}}}}$	3.06
Si${}_{{{{{{{{\rm{ads}}}}}}}}}^{{{{{{{{\rm{Nb}}}}}}}}}$	0.49

Results are for Si substituting on the Nb site, Si_Nb_, Si substituting on the Se site, Si_Se_, Si adsorbed above the hollow site, Si${}_{{{{{{{{\rm{ads}}}}}}}}}^{{{{{{{{\rm{hollow}}}}}}}}}$, Si adsorbed vertically above a Se atom, Si${}_{{{{{{{{\rm{ads}}}}}}}}}^{{{{{{{{\rm{Se}}}}}}}}}$, Si adsorbed vertically above a niobium site, Si${}_{{{{{{{{\rm{ads}}}}}}}}}^{{{{{{{{\rm{Nb}}}}}}}}}$, and and Si incorporated interstitially, Si_*i*_, in monolayer NbSe_2_.

**Fig. 4:**
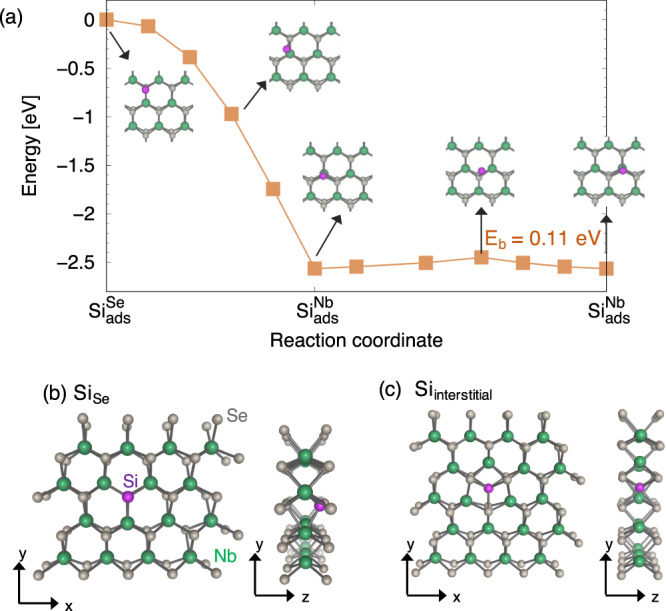
Adsorption and migration properties of Si in NbSe_2_. **a** Theoretical minimum-energy pathway for Si adatoms on NbSe_2_ to migrate from the Si${}_{{{{{{{{\rm{ads}}}}}}}}}^{{{{{{{{\rm{Se}}}}}}}}}$ metastable adsorption site to Si${}_{{{{{{{{\rm{ads}}}}}}}}}^{{{{{{{{\rm{Nb}}}}}}}}}$. The migration barrier to hop between Si${}_{{{{{{{{\rm{ads}}}}}}}}}^{{{{{{{{\rm{Nb}}}}}}}}}$ sites is 0.11 eV. The energies are reported with respect to the total energy of Si${}_{{{{{{{{\rm{ads}}}}}}}}}^{{{{{{{{\rm{Se}}}}}}}}}$. Schematic top view and side view of (**b**) Si substituted on the Se site, Si_Se_ and (**c**) Si incorporated interstitially within the same plane as the the Nb atoms, Si_*i*_.

If Si is deposited on the NbSe_2_ surface we find that it is likely to initially adsorb on the Si${}_{{{{{{{{\rm{ads}}}}}}}}}^{{{{{{{{\rm{Nb}}}}}}}}}$ site, not the Si${}_{{{{{{{{\rm{ads}}}}}}}}}^{{{{{{{{\rm{Se}}}}}}}}}$ site (which was implicitly assumed to be the most stable configuration for Si in the study by Zhao et al.^12^). The likelihood of Si remaining adsorbed on the NbSe_2_ surface is determined in part by the migration barrier of Si adatoms. We calculated the minimum energy pathway for a Si adatom to migrate from the metastable site, Si${}_{{{{{{{{\rm{ads}}}}}}}}}^{{{{{{{{\rm{Se}}}}}}}}}$, to the Si${}_{{{{{{{{\rm{ads}}}}}}}}}^{{{{{{{{\rm{Nb}}}}}}}}}$ adsorption site and find it to be barrier-less as illustrated in Fig. 4a. Subsequent hops between Si${}_{{{{{{{{\rm{ads}}}}}}}}}^{{{{{{{{\rm{Nb}}}}}}}}}$ sites occurs with a low migration barrier of 0.11 eV, which would render Si adatoms to be highly mobile even at low temperatures.

The low migration barrier of Si on the surface of NbSe_2_. makes it unlikely that Si adatoms will exist as isolated defects. Our formation energy calculations suggest that Si adatoms will likely be incorporated as Si_Se_ or as Si_*i*_, which are the two lowest energy configurations for Si that is not adsorbed on NbSe_2_. If there is a large concentration of Se vacancies, as we suggest are present in the study by Zhao et al.^12^, Si that is deposited on NbSe_2_ will migrate and substitute for the Se sites when it encounters a *V*_Se_. The STM images of Si deposited on NbSe_2_ (cf. Fig. S9a in ref. ^12^) provides us a clue that supports this assertion. The STM images show bright spots associated with Si residing at Se sites. Our formation energy calculations in Table 2 show that it is unlikely for Si to adsorbed above Se. Hence, the experiments by Zhao et al.^12^ unambiguously shows that Si atoms indeed substitute for Se. When Si substitutes for Se as illustrated in Fig. 4b, Si is bonded to three nearest-neighbor Nb atoms with a Si-Nb bond length of 2.615 Å, which is 4.5% shorter than the equilibrium Nb-Se bond length.

For higher concentrations of Si, once all of the Se vacancy sites are occupied we expect Si to incorporate as an interstitial where it sits in the same plane as the Nb atoms. In this configuration, Si_*i*_ displaces one of the Nb atoms that it is adjacent to and is six-fold coordinated by the Se atoms with a Si-Se bond length of ~2.60 Å, which is illustrated in Fig. 4c.

## Discussion

Here we propose a framework to understand how this seemingly diverse set of results on Si and S in NbSe_2_ can be used to understand the non-monotonic change of *T*_*c*_ in NbSe_2_ observed by Zhao et al.^12^, without invoking multifractality. Since the formation energy of Se vacancies in NbSe_2_ is low, Se vacancies are likely to exist in considerable concentration in as-grown NbSe_2_, which is consistent with experimental observations. We have previously shown, using first-principles calculations, that *V*_Se_ in NbSe_2_ is a magnetic point defect that results in a finite magnetization of ~0.6 *μ*_B_^26^. This finite magnetization spans a length scale of up to 15 Å and is likely to have an easy axis along $\hat{z}$. Hence, in the Ising superconductor NbSe_2_ they will be pair-breaking^26^ and suppress *T*_*c*_, as in a regular *s*-wave superconductor^31^. This is consistent with measurements on ML NbSe_2_, where low values of *T*_*c*_ are found in samples where the residual resistivity ratio is low^25^. Our calculations show that the substitution of S on the Se site in NbSe_2_ is energetically favorable for all S compositions. Hence, during the growth of NbS_x_Se_2−x_ we expect S to occupy the sites of missing Se atoms up to a critical S composition. This would lower the concentration of pair-breaking *V*_Se_ defects and lead to an increase in *T*_*c*_. This immediately explains why the putative multifractal behavior was observed in NbSe_2_ samples with suppressed *T*_*c*_, compared to the samples in the literature with lower defect concentrations, which is correlated with higher *T*_*c*_.

We also anticipate the presence of Se vacancies to play a key role when Si is deposited on NbSe_2_^12^. The low migration barrier that we calculate for Si adatoms implies that the Si adatoms that are initially adsorbed vertically above the Nb site will migrate and occupy *V*_Se_ or Si_*i*_ sites. As *V*_Se_ sites are occupied by Si, the pair-breaking effect of *V*_Se_ will decrease and we expect *T*_*c*_ to increase as was indeed observed in by Zhao et al.^12^ until all of the *V*_Se_ sites are occupied. In the experiments where fractal superconductivity was invoked^12^ we expect this to occur up to a Si coverage of ~0.05 Si atoms per NbSe_2_ formula unit, which is where the peak *T*_*c*_ in the NbSe_2_ samples with Si occurs (Fig. 1). Beyond this concentration, we expect the Si atoms to incorporate interstitially within the NbSe_2_ lattice as illustrated in Fig. 4c.

While the increase in *T*_*c*_ in the NbSe_2_ samples with S and Si can be understood by considering the role of *V*_Se_ the subsequent reduction in *T*_*c*_ at higher concentrations of S and Si can have different origins. One possible consideration is the role of ionized impurity scattering. However, experimentally, there is no indication of ionized impurity scattering in the NbS_x_Se_2−x_ alloys since *T*_*c*_ decreases monotonically for *x* ≥ 0.4 (Fig. 1) and does not exhibit any convex variations of *T*_*c*_ with *x*.

We also find that both NbSe_2_ and NbS_2_ host strong spin fluctuations at all wave vectors. First-principles calculations on monolayer NbSe_2_ suggest that spin fluctuations play a role in suppressing *T*_*c*_ when compared to the *T*_*c*_ obtained by electron-phonon coupling alone (S. Das, H. Paudyal, D. Agterberg, E.R. Margine and I. I. Mazin, Electron-phonon coupling and spin fluctuations in Ising superconductor NbSe_2_, Unpublished, 2022). However, for finite S compositions in NbS_x_Se_2−x_ this tendency towards magnetism is weakened, which favors superconductivity. This reduction in the proximity to magnetism competes with a reduction in *N*(E_F_) of the alloys compared to NbSe_2_ and NbS_2_, which would decrease the electron-phonon coupling constant, *λ*_*ep*_, and weaken superconductivity. We can estimate the sign of the net effect using the general expression derived in ref. ^32^, under a simplifying assumption that the spin-fluctuations and phonons have comparable frequencies. Then $d\log {T}_{c}/d\log {\lambda }_{ep}\propto {\lambda }_{ep}+2{\lambda }_{ep}{\lambda }_{sf},$ and $-d\log {T}_{c}/d\log {\lambda }_{sf}\propto {\lambda }_{sf}+2{\lambda }_{sf}{\lambda }_{ep}.$ The DOS is reduced by ≈10% between the end composition and the midpoint. In contrast, the tendency to magnetism, as measured by the spin-spiral energy gain, decreases by a factor of 2, between the end composition and the midpoint. The latter is expected to be more important, at least for low concentrations of S. We reiterate, based on our considerations of the pair-breaking effect of selenium vacancies and the changes in the electronic structure due to alloying with S, the presence of *V*_Se_ that are subsequently occupied by S, plays the primary role in leading to the non-monotonic change in *T*_*c*_ in the NbS_*x*_Se_(2−*x*)_^12^. We expect the changes in Δ*E*_spiral_, *χ* and *N*(*E*_*F*_) to have a secondary effect on *T*_*c*_.

Silicon incorporation in NbSe_2_ leads to a more pronounced suppression of *T*_*c*_ compared to S. This suppression is unlikely to be due to ionized impurity scattering as evidenced by the linear change in *T*_*c*_ with Si content. We suggest Si incorporated interstitially leads to doping of NbSe_2_ that decreases *T*_*c*_.

Taken together, these properties of S and Si in NbSe_2_ collectively imply that the non-monotonic dependence of *T*_*c*_ on S and Si content^12^ is not sufficient proof of fractal superconductivity, but likely has a rather prosaic origin: it is S or Si occupying a high concentration of Se vacancy sites (thus decreasing the concentration of pair-breaking defects). For the case of alloying with S this is also accompanied by a strong reduction of the tendency to magnetism as the S concentration increases from 0 to ~0.5. This effect overlaps with the general weaking of the electron-phonon matrix elements, as evidenced by the smaller coupling constant in NbS_2_ compared to NbSe_2_, despite their similar *N*(E_F_) (S. Das, H. Paudyal, D. Agterberg, E.R. Margine and I. I. Mazin, Electron-phonon coupling and spin fluctuations in Ising superconductor NbSe_2_, Unpublished, 2022)^33,34^. For the case of Si in NbSe_2_ we expect that when Si is incorporated interstitially once all of the *V*_Se_ sites are occupied, Si_*i*_ will act as a source of doping and decrease *T*_*c*_.

We have discussed two possible mechanisms that can lead to an increase in *T*_*c*_ when S or Si is incorporated in NbSe_2_; (i) the presence of Se vacancies that are pair-breaking defects and (ii) a reduction in the tendency to magnetism when NbSe_2_ is alloyed with S. Of these two mechanisms we suggest the presence of Se vacancies is likely playing a larger role. If the concentration of Se vacancies were reduced their pair-breaking effect would be suppressed. This would lead to a higher value of *T*_*c*_ at *x* = 0 and the non-monotonicity that was observed would be less pronounced. Indeed, if we linearly extrapolate the *T*_*c*_(*x*) data for *x* ≥ 0.4 to *x* = 0, in the NbSe_2_ samples alloyed with S we get a *T*_*c*_ of NbSe_2_ that ranges from 3.35 K to 4.2 K, as illustrated in Fig. 1. We find a similar *T*_*c*_ of ~4 K for NbSe_2_ if we linearly extrapolate *T*_*c*_ for *y* ≥ 0.05 for the NbSe_2_ samples with Si. Furthermore, this is consistent with optimizing growth conditions, which leads to a suppression in the concentration of selenium vacancies, which in turn leads to higher values of *T*_*c*_ in monolayer NbSe_2_^35^.

Our results, when analyzed in the context of recent studies that have asserted the presence of fractal superconductivity when S and Si are incorporated in NbSe_2_^12,17^, leads us to conclude that multifractality isn’t the only mechanism that can lead to non-monotonic changes in *T*_*c*_. The following key factors emerge from our calculations: (1) the low formation energy of Se vacancies that are magnetic pair-breaking point defects, (2) the stability of NbS_x_Se_2−x_ alloys across the entire composition range with respect to decomposition into the parent compounds, (3) the low migration barrier for Si adatoms on NbSe_2_ and the low formation energy for Si substitution on the Se site in NbSe_2_, (4) the reduction in the density of states at the Fermi level as a function of alloy content in NbS_x_Se_2−x_ and (5) a reduction in the proximity to magnetism in NbS_x_Se_2−x_ alloys compared to NbSe_2_ and NbS_2_.

These results suggest that as-grown NbSe_2_ hosts a large concentration of pair-breaking Se vacancies that upon alloying are occupied by sulfur or silicon atoms. This leads to an increase in *T*_*c*_ up to a critical composition where the concentration of sulfur or silicon is equal to the concentration of Se vacancies that are present during the growth. For the case of alloying with sulfur, *T*_*c*_ monotonically decreases once sulfur occupies all of the Se vacancy sites, reflecting a general weakening of the electron-phonon matrix elements toward NbS_2_. For high concentrations of silicon in NbSe_2_ we find silicon is also likely to incorporate interstitially, where it would act as a dopant and lead to a reduction in *T*_*c*_. These two distinct regimes manifest in a non-monotonic change in *T*_*c*_. Based on this scenario we also predict that if an experiment similar to that by Zhao et al.^12^ is performed on samples with a lower concentration of selenium vacancies and a higher initial *T*_*c*_, the non-monotonic change in *T*_*c*_ will be less pronounced or even fully suppressed. Given that disorder-induced non-monotonic changes in *T*_*c*_ have been observed in other transition metal dichalcogenide alloys due to isovalent substitution^36–38^, we expect our findings to open new avenues for investigation in this broad class of materials.

## Methods

Our calculations are based on density functional theory within the projector-augmented wave method^39^ as implemented in the VASP code^40,41^ using the generalized gradient approximation defined by the Perdew-Burke-Ernzerhof (PBE) functional^42^. We found it is essential that Nb 5*s*^1^, 4*s*^2^, 4*p*^6^, 4*d*^4^ electrons and Se 4*s*^2^, 4*p*^4^ electrons are treated as valence. All calculations use a plane-wave energy cutoff of 400 eV. We use a (18 × 18 × 1) Γ-centered *k*-point grid for the monolayer structure when performing structural optimization and calculating the electronic structure. The cell shape and atomic positions of each structure was optimized using a force convergence criteria of 5 meV/Å. All of the structures were optimized in the non-magnetic state. We verified that optimizing the structures in the spin spiral state leads to minor differences in the lattice parameters.

For the calculations of chalcogen vacancies we use a (10 × 10 × 1) supercell of ML NbSe_2_ and NbSe_2_. To simulate a chalcogen vacancy we remove a single chalcogen atom (S atom in NbS_2_ and Se atom in NbSe_2_), relax all of the atomic coordinates and determine the total energy. The formation energy, of for example, a Se vacancy, *V*_Se_ in NbSe_2_ is defined as:

1 $${E}^{f}({V}_{{{{{{{{\rm{Se}}}}}}}}})={E}_{{{{{{{{\rm{tot}}}}}}}}}({V}_{{{{{{{{\rm{Se}}}}}}}}})-{E}_{{{{{{{{\rm{tot}}}}}}}}}({{{{{{{{\rm{NbSe}}}}}}}}}_{2})-{\mu }_{{{{{{{{\rm{Se}}}}}}}}}$$

where *E*^*f*^(*V*_Se_) is the formation energy of the Se vacancy, *E*_tot_(*V*_Se_) is the total energy of the NbSe_2_ defect supercell with a Se vacancy, *E*_tot_(NbSe_2_) is the total energy of the pristine NbSe_2_ supercell, and *μ*_Se_ is the chemical potential of Se. For the calculation of Si in NbSe_2_ we use an orthorhombic supercell with 64 NbSe_2_ formula units. We use the formation enthalpy of SiSe_2_ as the limiting phase for our formation energy calculations of Si in NbSe_2_. All of the defect calculations were performed with a (3 × 3 × 1) *k*-point grid. The theoretical minimum energy pathway for adatom migration on NbSe_2_ was calculated using the using the nudged elastic-band method^43^.

For the calculations of the alloy properties with sulfur we consider two approaches; the virtual crystal approximation (VCA) and explicit supercell calculations using either a (4 × 1 × 1) and a (4 × 4 × 1) supercell that is constructed from the unit cell of the ML structure. For each alloy supercell we consider different arrangements of the S and Se atoms for compositions corresponding to *x* = 0.125, 0.25, 0.5, 1, and 1.5, and relax all of the atomic positions. The *k*-point grid for structural relaxation of each supercell is scaled with respect to the (18 × 18 × 1) Γ-centered *k*-point grid we use for calculations of the unit cell.

To determine the thermodynamics of alloy formation we calculated the formation enthalpy, Δ*H*(*x*), as a function of sulfur content, *x*, using the (4 × 4 × 1) supercell. Δ*H*(*x*) is defined as:

2 $${{\Delta }}H(x)=E(x)-xE({{{{{{{{\rm{NbS}}}}}}}}}_{2})-(1-{{{{{{{\rm{x}}}}}}}}){{{{{{{\rm{E}}}}}}}}({{{{{{{{\rm{NbSe}}}}}}}}}_{2})$$

where *E*(*x*) is the total energy of the alloy supercell with sulfur content, *x*, *E*(NbS_2_) is the total energy of the NbS_2_ supercell and *E*(NbSe_2_) is the total energy of the NbSe_2_ supercell.

We varied the lattice parameters for each alloy configuration linearly as a function of sulfur content in accordance with Vegard’s law and then relax all of the atomic coordinates. For a given sulfur content, *x*, the in-plane lattice constant, *a*(NbS_x_Se_2−x_) was varied as *a*(NbS_x_Se_2−x_) = $x{a}_{{{{{{{{{\rm{NbS}}}}}}}}}_{{{{{{{{\rm{2}}}}}}}}}}$ + $(2-x){a}_{{{{{{{{{\rm{NbSe}}}}}}}}}_{{{{{{{{\rm{2}}}}}}}}}}$, where ${a}_{{{{{{{{{\rm{NbS}}}}}}}}}_{{{{{{{{\rm{2}}}}}}}}}}$ is the in-plane lattice parameter of bulk NbS_2_ and ${a}_{{{{{{{{{\rm{NbSe}}}}}}}}}_{{{{{{{{\rm{2}}}}}}}}}}$ is the in-plane lattice parameter of bulk NbSe_2_. We verified the accuracy of Vegard’s law for a subset of alloy structures by allowing the lattice parameters and atomic positions to relax. In all cases, the variation of the in-plane lattice parameters was linear (Supplementary information).

To calculate the spin spiral energies we used the generalized Bloch theorem formalism^44^ as implemented within VASP. We use a dense (36 × 36 × 1) Γ-centered *k*-point grid for the unit cell. We determine the energy difference between the spin spiral state with respect to the non-magnetic state, Δ*E*_spiral_, which is defined as Δ*E*_spiral_ = *E*(**q**) − *E*(**q** **=** **0**) where *E*(**q**) is the total energy of the unit cell with spin spiral wavevector **q** and *E*(**q** **=** **0**) is the total energy of the non-magnetic unitcell.

To determine the ferromagnetic spin susceptibility, *χ* we used collinear fixed-spin moment (FSM) calculations (sometimes referred to as the constrained local moments approach). In our collinear FSM calculations we constrain the magnitude of the magnetic moment on the Nb atom. Performing these calculations allows us to determine the change in energy with respect to the non-magnetic ground state as a function of the total magnetization, *m*. We then fit our results to an expansion of the total energy as a function of *m* to the following expression, *E*(*m*) = *a*_0_ + *a*_1_*m*^2^ + *a*_2_*m*^4^ + *a*_3_*m*^6^ + *a*_4_*m*^8^, where *E*(*m*) is the total energy for a given magnetization *m*, to determine the ferromagnetic spin susceptibility, *χ*. The spin susceptibility, *χ*, obtained from FSM calculations is sensitive to the choice in energy convergence threshold, and the number of magnetization values used in the fit to expansion in the total energy as a function of magnetic moment. We use an energy convergence threshold of 10^−8^ eV, and up to 50 magnetization versus energy points between 0  *μ*_*B*_ and 0.6 *μ*_*B*_ for all of the FSM calculations.

The results on the formation enthalpy of the alloys are obtained using a (4 × 4 × 1) supercell with tetrahedron smearing and a (9 × 9 × 1) *k*-point grid. The spin spiral energies, fixed spin moment calculations, and the density of states of the alloys are obtained using VCA calculations for sulfur contents that correspond to *x* = 0.5, 1, and 1.5. The VCA calculations use the same *k*-point grid as the unit cell calculations. The in-plane lattice parameters for the *x* = 0.5, 1, and 1.5 VCA calculations are scaled linearly according to Vegard’s law. Furthermore, we also interpolate the vertical Nb-chalcogen bond length along the *c*-axis for each VCA alloy calculation.

## Supplementary information

Supplementary Information

## Data Availability

The data that supports the findings of this study are available from the corresponding author upon reasonable request.
