# Supplementary material for: Effect of alloying in monolayer niobium dichalcogenide superconductors
Source: Nat Commun. 2022 May 2;13:2376. doi: 10.1038/s41467-022-29213-8 (PMC9061790; doi:10.1038/s41467-022-29213-8)
Supplement: Supplementary file 1 — Supplementary Information [file 41467_2022_29213_MOESM1_ESM.pdf]

Supplementary Information: Effect of alloying in monolayer niobium dichalcogenide superconductors

Darshana Wickramaratne
Center for Computational Materials Science, U.S. Naval Research Laboratory, Washington, DC 20375, USA

I.I. Mazin
Department of Physics and Astronomy, George Mason University, Fairfax, VA 22030, USA and
Quantum Science and Engineering Center, George Mason University, Fairfax, VA 22030, USA

SUPPLEMENTARY NOTE 1: ELECTRONIC AND MAGNETIC PROPERTIES OF MONOLAYER $NBSE_2$ AND NBS_2

Here, we present the band structure and density of states of ML $NbSe_2$ and NbS_2 obtained from our first-principles calculations. All of our calculations use the lattice parameters obtained from optimizing the structures within DFT. The calculated in-plane lattice constant of $NbSe_2$ is 3.449 Å and of NbS_2 is 3.32 Å.

The band structure of ML $NbSe_2$ and NbS_2 with spin-orbit coupling is illustrated in Figure 1.

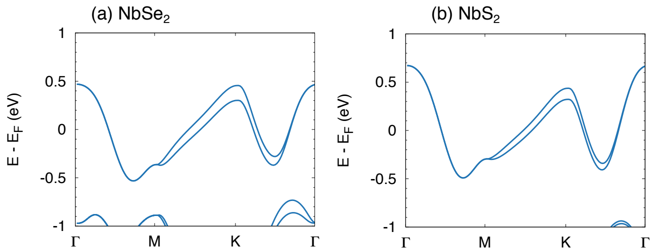

Supplementary Figure 1: Band structure with spin-orbit coupling of monolayer (a) $NbSe_2$ and (b) NbS_2.

From the band structures in Fig. 1 it is evident that the spin-orbit splitting is finite along the M-K-Γ line and is maximum at K. The spin-orbit splitting at K in $NbSe_2$ is $\sim$ 150 meV and in NbS_2 it is $\sim$ 115 meV.

In Figure 2 below we present calculations of spin spiral energies with respect to the non-magnetic state of $NbSe_2$ and NbS_2 for **q** vectors up to (0.5,0).

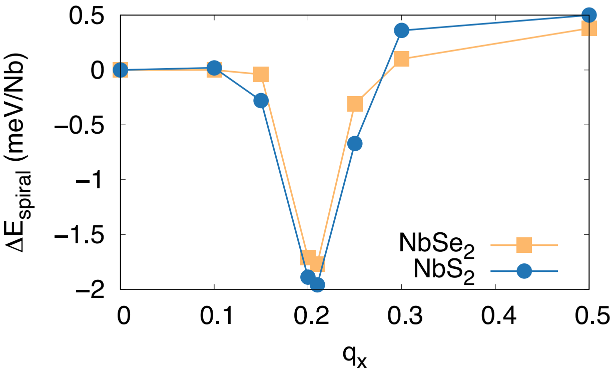

Supplementary Figure 2: Energies of the spin spiral state of monolayer $NbSe_2$ (orange squares) and NbS_2 (blue circles) with respect to the non magnetic state.

We find the spin spiral state of $NbSe_2$ to be stable at $\sim$(0.2,0). We also find a spin-spiral state to be stable at the same **q**-vector in NbS_2, with a slightly larger energy gain compared to $NbSe_2$.

We calculated the density of states of ML $NbSe_2$ and NbS_2 in the non-magnetic state and spin-spiral ground state. The results are illustrated in Fig. 3. In both materials the spin-spiral state leads to a reduction the DOS at the Fermi

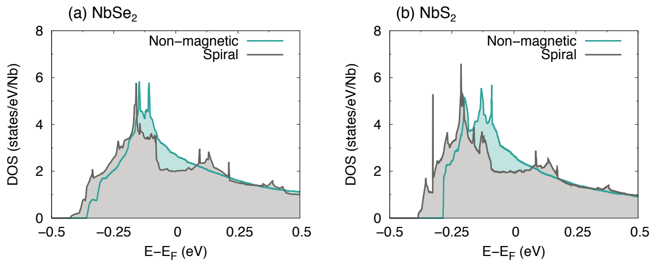

Supplementary Figure 3: DOS in the nonmagnetic and spin spiral state of monolayer (a) $NbSe_2$ and (b) NbS_2.

level, which is consistent with the spin spiral being the ground state. We note that the spin spiral calculations do not

include spin-orbit coupling. We verified that the magnitude of $N(E_F)$ in our non-collinear non-magnetic calculation and calculation with spin-orbit coupling is similar for both $NbSe_2$ and NbS_2.

SUPPLEMENTARY NOTE 2: STRUCTURAL PROPERTIES OF NBS_XSE_{2-X} ALLOYS

We compare the in-plane lattice constants of the NbS_xSe_{2-x} alloys obtained from our first-principles calculations with the experimental lattice constants reported.

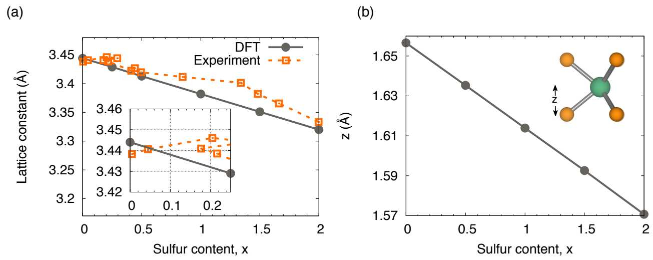

Supplementary Figure 4: (a) In-plane lattice constants of NbS_xSe_{2-x} alloys as a function of sulfur content, x. The calculated lattice constants (circles) are compared with the experimentally reported in-plane lattice constants (squares). (b) Change in vertical distance between the Nb and chalcogen atom along the c-axis as a function of alloy content. The inset illustrates the definition of z.

Our calculations indicate that the lattice constants vary linearly as a function of increasing sulfur content, i.e they obey Vegard's law. We note that the experimental lattice constants for $0 \leq x \leq 0.2$ remain relatively unchanged as illustrated in the inset of Fig. 4.

In addition to changes in the in-plane lattice parameter as a function of alloying it is also important to account for changes in the height of the Nb-chalcogen atoms, z, along the c-axis as a function of alloy content. We find z changes from 1.656Å in $NbSe_2$ to 1.571Å in NbS_2. In our VCA calculations we linearly interpolate the value of z between the Nb and chalcogen atom as illustrated in Fig. 4(b).
